# Supplementary material for: Chromothripsis during telomere crisis is independent of NHEJ, and consistent with a replicative origin
Source: Genome Res. 2019 May;29(5):737–49. doi: 10.1101/gr.240705.118 (PMC6499312; doi:10.1101/gr.240705.118)
Supplement: Supplemental Material [file supp_gr.240705.118_Supplemental_file_1.zip › contigs/annotated_contigs/DB49/contig.3.DB49_length_789_mean_cov_15.7148288973.docx]

**DB49_length_789_mean_cov_15.7148288973**

```
GATCC|GCCCGCCTCAGCTTCCCAAAGTGTGAGGATTACAGGGGTGAGCCACTGCACCCGGCTATAAATAGTCTCTTAATAGATTTCCT
      >chr5:31906276-31906723 - E=7e-247 p=0e+00
ACATCATTGATAAAAACCAAACTAAGGCCGCGCATGGTGGCTGACACCTGTAATCCTCACACTATGGGAGGCTGAGACGGGCTGATTGC

TTGAGCCCAGGAGTTCAAGACTAGTCTGGGTAACATGGCGAAACCCCGTCTCTACCAAAAATACAAAAACTGAGGTGGGAGGATCACCT

GAGCCTGGGGAGGTCAGGGTTACCACTGCACTCCAGCCTCTGCAACAGGGTGAGACCCCATCTCAAAAACAACAACAACAACAACAAAA

TCAACGAACATTTGGACATCGGTAACATGAAGTATATTTTACTTGTGTGGTATCTCAGTTAAAATTACTTCAATTTCAGGCCAGGTGTG

GTG|GC|TCATGCCTGTAATCCCAGCACTTTGGGAGCCCAAGGCGGGTTGTCACTTGAGTTCAGGAGTTTGGACCAACTTGGCCAACAT
    >chr12:101738734-101739009 - E=1e-50 p=0e+00
AGTAAAACCCCATCTCTACTAAAAATACAAAAATTAACTGGGCGTGGTGGCATGTACCTGTAATCCCGGCTACTTGGGAGTCTGAGGCT

GGAGAATAGCTTGAACCTGGGAGGCAGAAGTTGCAGTGAGCCAAGAACACACCACTCCACTCCAGCCTGGGTGACAGAGTGAGACTCCA

TCTCAAAAAAA|T|AAAATAAGGCTGGGCGTGGTGGCTTATGCCTGTAATCCCTG|TAATCAATGGAGATTGAGCCCATTCTG             >chr14:91545513-91545554 - E=8e-10
```
